# Role of New Nature Reserve in Assisting Endangered Species Conservation - Case Study of Giant Pandas in the Northern Qionglai Mountains, China

**DOI:** 10.1371/journal.pone.0159738

**Published:** 2016-08-17

**Authors:** Tian-Pei Guan, Jacob R. Owens, Ming-Hao Gong, Gang Liu, Zhi-Yun Ouyang, Yan-Ling Song

**Affiliations:** 1 Ecological Security and Protection Key Laboratory of Sichuan Province, Mianyang Normal University, Mianyang, China; 2 Chengdu Research Base of Giant Panda Breeding, Chengdu, China; 3 Research Institute of Wetland, Chinese Academy of Forestry, Beijing, China; 4 Research Center for Eco-Environmental Sciences, Chinese Academy of Sciences, Beijing, China; 5 Institute of Zoology, Chinese Academy of Sciences, Beijing, China; Università degli Studi di Napoli Federico II, ITALY

## Abstract

The creation of nature reserves is the most direct way to save endangered species populations and their habitat. Development of the giant panda (*Ailuropoda melanoleuca*) nature reserve network in China was initiated in the 1960s, though the effort to create new reserves boomed considerably after the year 2000. Given this rapid development of protected areas in panda habitats, and the potential conflicting interests between conservation administrations and local economic development, it is essential to assess the role of new nature reserves in the overall giant panda conservation effort and reserve network. We utilized data from national giant panda surveys conducted in 2000 and 2012 to compare the size, spatial use, and distribution of panda populations, as well as the habitat suitability and connectivity in the Northern Qionglai Mountains between the two survey years. Our results show that although the total giant panda population in the study area did not change remarkably, local changes did occur. Most notably, the population in Wolong Nature Reserve declined by 27.3% (N = 39) and the population in Caopo Nature Reserve increased by 71.4% (N = 29) over the 12-year study period. We also found habitat suitability and availability decreased in both Wolong (12.4%) and Caopo (7.4%), but that the relative density of giant pandas declined (19.2%) and increased (84.6%) at each site, respectively. The distance between centers of high IUA were more distant in 2012 (14.1±1.9km) than that in 2000 (6.1±0.9km; t = -7.4, df = 5, p = 0.001), showing a scattered spatial pattern. Habitat availability decreased by 42% within the corridor between the two reserves, however panda occurrences in the corridor increased 24.6%. Compared to the total number of encounters, the proportion of the corridor increased 45.76%. Our results show the importance and success of the newly established Caopo to the conservation of giant pandas, and how crucial it is to identify and repair reserve corridors. Furthermore, we propose criteria for future nature reserve network management and investment, which is applicable for other endangered species conservation practices.

## Introduction

The creation of protected areas, including nature reserves and national parks, is one of the most effective measures to conserve endangered species, as well as the habitats and ecosystems necessary for their survival [1, 2]. Many nature reserves with long histories, such as Yellowstone National Park in the U.S.A. and Masai Mara National Reserve in Kenya, have contributed notably to the conservation of the wildlife and habitats within their boundaries [3, 4]. Beyond these well-established nature reserves, developing countries worldwide increasingly seek to mitigate biodiversity declines by creating their own reserves [5, 6]. However, considerable conflicts often exist between the conservation of natural resources and their exploitation for economic development [7]. Given that these resources are often important to both wildlife and humans, it is essential to evaluate the performance of newly established reserves as nature reserve networks expand. Such information can be used to increase the efficiency of natural resource management, and limit the amount of land, funding, and other resources that are poorly used.

Giant pandas (*Ailuropoda melanoleuca*) are a conservation flagship species and one of the most widely recognized icons of conservation worldwide. In response to the recognition of their highly endangered status and rarity in the wild, China began creating giant panda nature reserves in 1960’s [8]. Since the year 2000, the effort to create new reserves in the giant panda nature reserve network, and reduce the existing high isolation and fragmentation of giant panda habitats, has increased considerably [6]. Prior to the turn of the century, there were 40 giant panda nature reserves, covering 2,175,780 ha. In the subsequent 12 years, 27 additional reserves were created, resulting in a total of 3,356,205 ha of land protected for giant pandas (State Forestry Administration of China, SFA Unpublished data). Given the rapid development of new nature reserves over this 12 year period (2000–2012) and the simultaneous economic growth in China, there is widespread public interest in assessing the performance of these reserves in the effort to conserve giant pandas [9]. Recognition of the role of these new nature reserves would be a crucial step to improve overall success of giant panda nature reserve network. However, to our best knowledge, the performance of new nature reserves has yet to be assessed.

In theory, the additional protected land afforded by new nature reserves should enable local giant panda populations to stabilize or grow by providing new habitat or refuge. To adequately assess the role of new established nature reserves requires several types of data, including the (1) size and proportion of habitat provided by the new protected area, (2) habitat connectivity of the area between existing and new nature reserves, (3) variation in population size and (4) spatial distribution over time (through GPS, molecular, or other available data sources).

A massive earthquake in 2008 effected the entire giant panda distribution in the northern Qionglai, resulting in serious ecological and socioeconomic consequences (i.e. forest destruction and landslides)[10]. The epicenter of the earthquake was only 3 km away from Wolong Nature Reserve, and resulted in a 6.5% reduction of suitable habitat to giant pandas within the reserve [11]. Following the earthquake, reconstruction programs threatened the recovering habitat with additional habitat loss and high levels of disturbance [8, 12]. As a member of the nature reserve network in northern Qionglai, Caopo Provincial Nature Reserve (Caopo) was created on the north edge of Wolong in 2000. Due to the influence of this natural event and subsequent anthropogenic disturbances, changes in critical habitat might have accelerated or triggered observable fluctuations in giant panda populations or their habitat use, and therefore provide an opportunity to assess this new nature reserve.

Our goal in this study was to assess the potential role of recently established nature reserves in contributing to the existing giant panda conservation network and population status at a regional scale. Although genetic and GPS collar data would be powerful in providing direct evidence of changes in the local population dynamics over the study period (e.g. immigrant or dispersal), no such data currently exists in the study area. Consequently, we assess the performance of Caopo only with reference to the rate of suitable habitat loss, proportion of suitable habitat available, habitat connectivity to Wolong, and the size and spatial distribution pattern of the populations pre- and post-disturbance. If Caopo played a positive role in conserving giant panda in northern Qionglai, we will observe high proportion suitable, well-connected habitat in Caopo both before and after the disturbance, as well as an increased population size following the disturbance. As a consequence, we expect to find a stable or increased population at regional scale.

## Methods

### Study area

**Fig 1:**
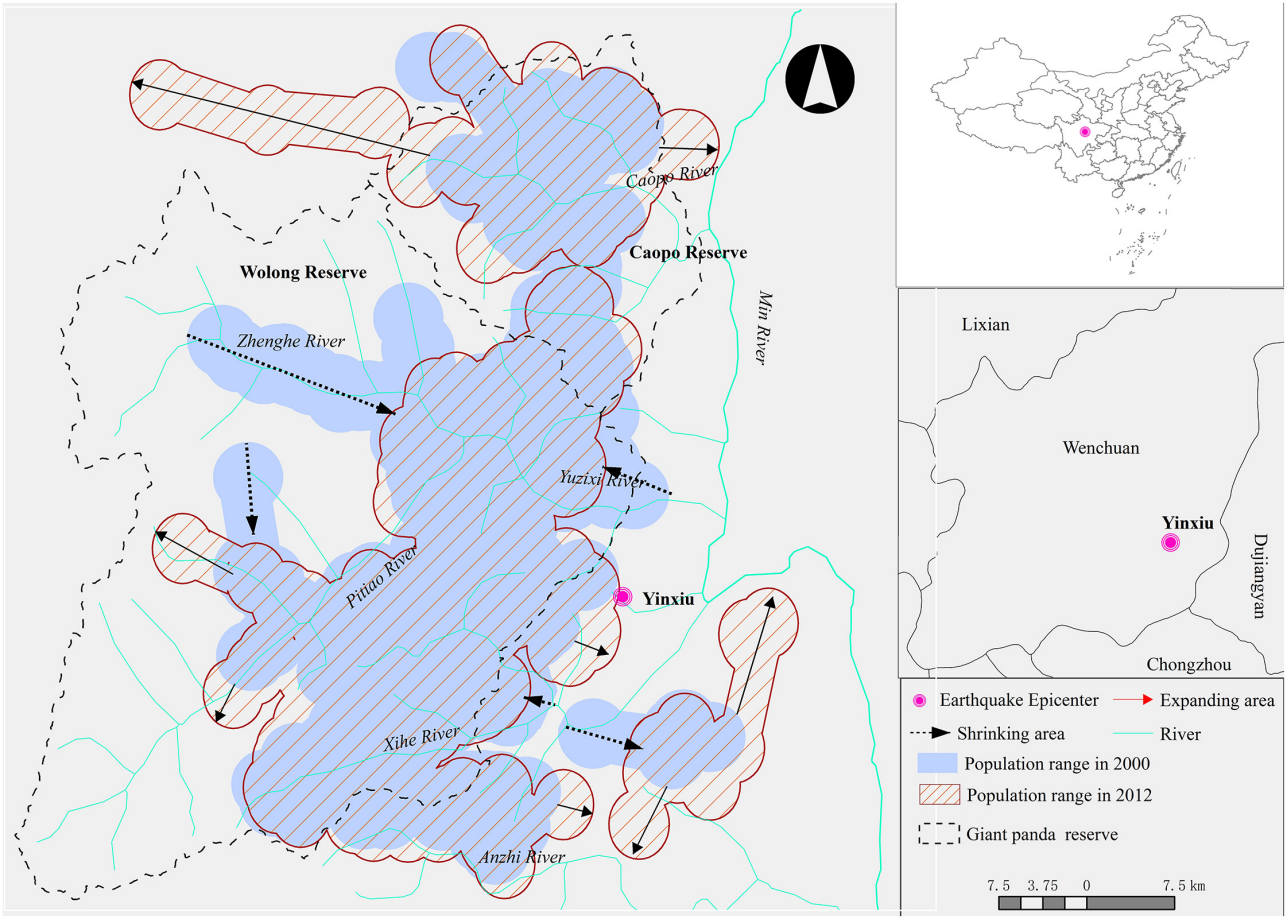
Giant panda habitat suitability, population distribution and range in the Northern Qionglai Mountains in 2012, Southwest of China.

Our study area is located in the northern Qionglai of southwestern China, covering all of Wenchuan and part of Lixian, Dujiangyan and Chongzhou counties (102°49’51”—103°39’19” E, 30°44’02”—31°28’47” N; Fig 1). The area is on the west side of the Min River, which is composed of several smaller tributaries, including the Caopo, Yuzixi, Zhenghe, Xihe and Anzihe, and is an important tributary of the Yangtze River. Located in the transition zone between the Tibetan Plateau and the Sichuan Basin, the elevation of the study area ranges from < 500 m to > 6,200 m asl, and contains multiple climatic zones and high biodiversity. In addition, this is a core habitat for giant panda and other rare species[13], and consists of two nature reserves, Wolong (200,000ha) and Caopo (55,612ha).

Wolong was established on the northern part of the Qionglai Mountains (Qionglai) in 1963, and was the first area started giant panda conservation projects in 1980[14] [15], containing more than 8% of the remaining wild giant pandas [16]. As one of the first giant panda nature reserve and the core giant panda habitat in Qionglai, Wolong remains a paradigm of giant panda conservation efforts. Caopo Provincial Nature Reserve (Caopo), is adjacent to the north edge of Wolong and constituting the northern distribution limit of giant panda in Qionglai, part of the Qionglai nature reserve network. At the landscape scale, Caopo and Wolong are isolated habitats, due to natural barriers that include high elevation mountain (>3900m asl) on the north, west and south, and a large river (Min River) on the east.

Both giant panda nature reserves follow a giant panda nature reserve management protocol to control human disturbance and implement a habitat monitoring program, which was initiated in 2003 [12]. There were no natural disturbances (fire, bamboo flowering, etc.) or giant panda mortalities reported from this monitoring program until the massive earthquake occurred in 2008. The epicenter of the Wenchuan earthquake was located in the town of Yingxiu, 3 km from the eastern border of Wolong (Fig 1). Landslides caused by the earthquake resulted in 29,419 ha of habitat loss within the reserve [13].

### Data source and survey protocol

Datasets used in this study were obtained from the Third and Fourth National Giant Panda Surveys (NGPS), which were conducted from May to July in 2000 and 2012, respectively, by the State Forestry Administration of the Peoples’s Repubilc of China. The Third NGPS [16] provided giant panda population data prior to the creation of Caopo, and the Fourth NGPS (State Forestry Administration of China, unpublished data) provided data following the both the creation of Caopo. During each NGPS, the same transect survey protocol was applied [16]. Line transect surveys were conducted within pre-defined 2 km^2^ survey grid cells that covered the entire known giant panda range and potential habitat. All fresh signs of giant pandas (i.e., fecal droppings) observed along each transect were recorded. Information recorded from each sign including its latitude, longitude, elevation, slope, vegetation type, and bamboo species present. Throughout the study area, there were 635 giant panda signs recorded from 1015 transects in the Third NGPS and 458 signs from 1076 transects in the Fourth NGPS. In addition, vegetation and bamboo in the study area were obtained from the two NGPS field surveys and satellite images, from Landsat 5 in 2000 and Spot5 in 2012, by using the maximum likelihood classification algorithm in supervised classification by Erdas 8.7 (Leica Geosystems GIS and Mapping, 2003, LLC, Atlanta, GA, USA).

Use of a 1:50,000 digital geographic map of the study area was approved by (and copied from) the Chinese Academy of Sciences (CAS), and data concerning landslides were provided by the Research Center for Eco-Environmental Sciences of the CAS. Disturbance data were collected via field investigations and interviews with residents from October to December 2013. Disturbances included the locations of tourist sites, hydropower stations, and mines. All geospatial data were analyzed by ArcGIS10.0, UTM and WGS84 coordinate system.

### Habitat Suitability Modelling

To identify suitable habitat, a maximum entropy modelling technique was applied using MaxEnt software [17]. This technique has been extensively used for habitat suitability modelling with presence-only data [18]. Prior to the selection of the candidate variables, the entire study area was divided into 1 km^2^ grid cells for further zonal function in ArcGIS. Next, variables that were known or presumed to be important to giant panda habitat utilization were converted into raster form for further value extraction within each grid cell (the original resolution of geographic dataset was 30m×30m) and in relation to presence points. Giant panda habitat use is traditionally defined as the degree of preference on habitat factors including terrain, land cover, and human disturbance [19,20]. Two types of candidate variables were assessed, including environmental variables (mean elevation, standard deviation of elevation, elevation range, total forest cover, conifer forest cover, mean slope, standard deviation of slope, and distance to river) and disturbance variables (distance to road, distance to hydropower station, and distance to human residences). To compare the habitat suitability in 2000 and 2012, the giant panda presence data and environmental data in 2000 was used to build a suitable model (10% of the locations were randomly selected and used as model training data), which was then projected onto the environmental data collected in 2012. Individual habitat suitability maps were derived for each survey year. By using these suitable habitat prediction maps, habitat suitability was divided into three levels, including unsuitable (<0.5), sub-suitable (0.5–0.75), and suitable habitat (>0.75). The size and percent of each suitability level within the total area were calculated. Performance of the models were evaluated by the area under curve (AUC), and the models were considered reliable when AUC value >0.75[21]. The relative contributions of the variables were confirmed by their permutation importance values in the analysis of variable importance table [22]. Habitat loss ratio (HLR) and available habitat ratio (AHR) were calculated based on area lost or available habitat area divided by the nature reserve area respectively. Based on suitable habitat maps, we could also identify the area of habitat well connected at the border between the two nature reserves to evaluate the degree of connectivity.

### Population distribution and relative density

Maps of the giant panda population distributions were created based on panda signs recorded during the two NGPSs. As a primarily solitary animal [14], the giant panda has a relatively exclusive core range, and its signs (i.e., fecal droppings, dens or sleeping sites) are effective indicators of activity range. Previous studies in Wolong Nature Reserve have estimated the diameter of the giant panda home range ranges from 1km to 3km [14, 19]. To provide an estimate of the possible area utilized by each panda encountered in the survey, panda signs were used to denote the center of a circle with a radius of 3 km, using the buffer function. This circle represented the maximum likely area that a giant panda might utilize (based on the 3 km diameter maximum), and allows for signs encountered at the very extremes of the individual home ranges. Polygons were created based on these circles to establish the maximum population distribution. Population size for the distribution range in the study areas were based on the results of NGPS in 2000 [16] and 2012 (State Forestry Administration of China unpublished data). The population size results of the NGPS’s were also used to calculate the density of individuals in the reserves based on the area of available habitat (sub-suitable and suitable) that they could utilize. The relative densities were calculated separately for Wolong and Caopo in both 2000 and 2012.

### Spatial utilization pattern of population

An increased density of giant panda signs encountered at a given location during a field survey represents a higher utilization intensity of giant panda population in that area. To assess differences in utilization density between the two survey periods, density maps of giant panda signs were developed using the kernel density estimation (KDE) function. Both density maps in 2000 and 2012 were divided into three levels (low, medium, high). The centroids of higher intensity (medium and high density) areas, which act as the centers of population-wide spatial utilization, were extracted. Using these results, the spatial patterns of pandas in the Qionglai were compared before and after new nature reserve was created, based on the centroids of the medium and high intensity utilization areas (IUA), using average nearest neighbor function. Finally, the distances between adjacent centroids of high IUA were calculated to reveal detailed shifts in spatial utilization patterns over the study period.

If there is suitable habitat in the region near the boundary of the two reserves, we will analyze the changes in corridor use between the two NGPS by comparing giant panda occurrence based on habitat suitability modelling. Corridor utilization was calculated in two ways (1) the proportion of locations found within the corridor and (2) the density of locations found within the corridor.

## Results

### Habitat suitability changes

**Fig 2:**
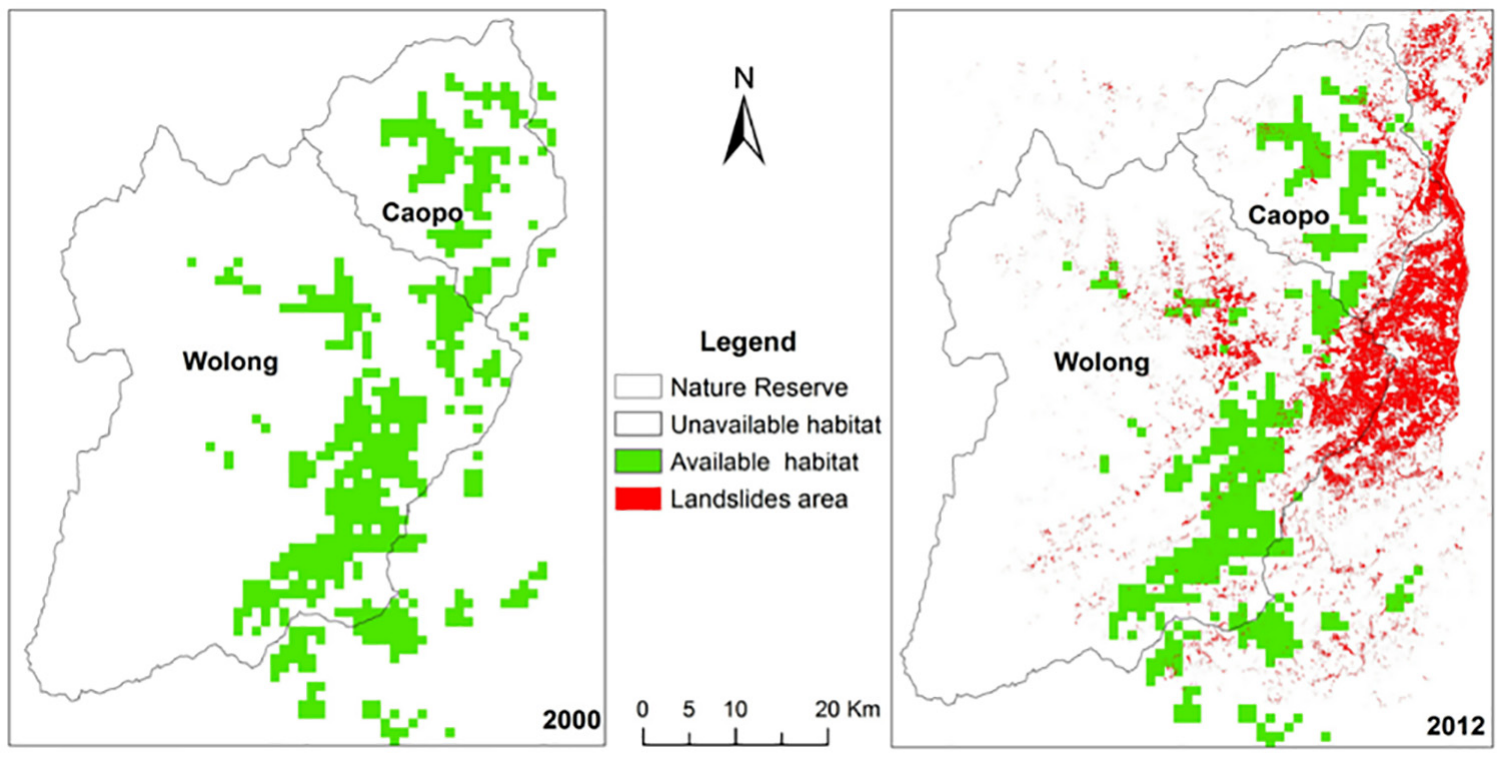
Giant panda available habitat distribution in 2000 and 2012 within Northern Qionglai Mountains, China.

The projected giant panda habitat model for the 2012 survey data resulted in AUC values of 0.94 with the model training data and 0.89 with the test data, indicating reliable prediction ability. Based on the model estimates, the area of available habitat (sub-suitable and suitable) in 2000 was 55,078 ha in Wolong (AHR = 27.54%) and 21,371 ha in Caopo (AHR = 38.43%). In 2012, available habitat decreased in Wolong by 12.4%(HLR) to 48,257 ha (AHR = 24.12%), and in Caopo by 7.4% (HLR) to 19,779 ha (AHR = 35.56%) (Fig 2). In the corridor area between Caopo and Wolong the amount of suitable habitat available declined by 42.2% throughout the study period, from 4,500 ha in 2000 to 2,600 ha in 2012 (Fig 2).

According to the permutation contribution of all variables in the model, mean elevation (34.4%) and forest cover (14.3%), and the proximity to residents (8.3%), hydropower stations (5.5%), and roads (5.1%) were main impact factors determining giant panda habitat suitability.

### Population distribution and relative density

The distributional range of giant pandas over the entire study area increased by 3.8% within 12 years, from 170,656 ha in 2000, to 177,211 ha in 2012 (Fig 1). A total of 190 giant pandas were identified within the study area in 2000. This included 143 in Wolong, 28 in Caopo, and 19 outside of the nature reserves. In 2012, 183 giant pandas were identified in study area, including 104 in Wolong, 48 in Caopo, and 31 outside of the nature reserves. Thus, over the 12-year study period the population of Wolong declined by 27.3%, while the Caopo population increased by 71.4%. As a result, the relative density of giant pandas in Wolong declined by 19.2%, from 2.6×10^−3^ ind/ha to 2.1 ×10^−3^ ind/ha. During this period the relative density of pandas in Caopo increased by 84.6%, from 1.3 ×10^-3^ind/ha to 2.4 ×10^−3^ ind/ ha. However, Comparing with the population change in Caopo and Wolong, the population size of entire northern Qionglai was relative stable, with only slightly decline (3.6%).

### Spatial utilization pattern of population

**Fig 3:**
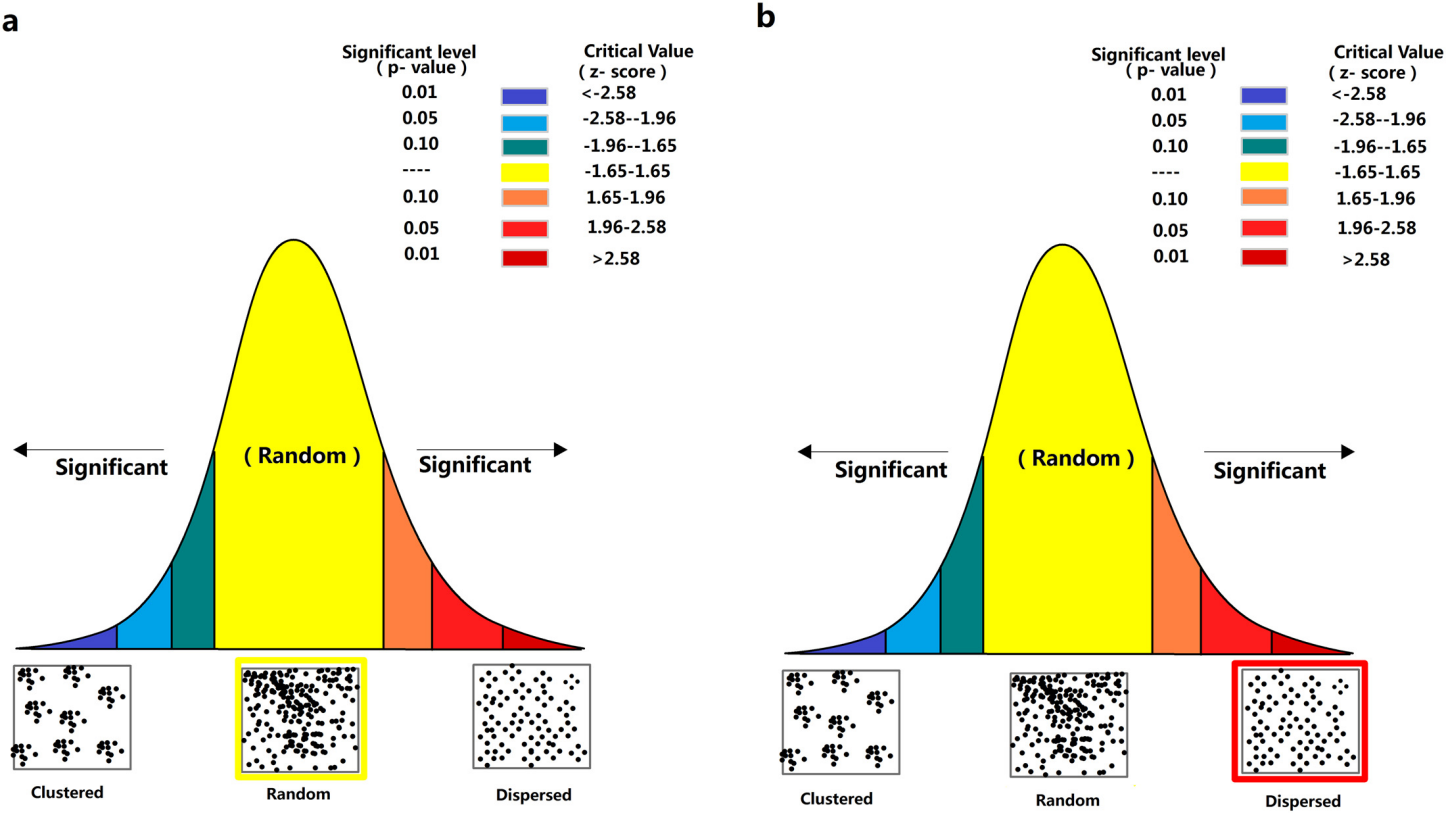
Spatial pattern analysis of medium intense utilization area (IUA) in 2000 (a) and 2012 (b). The center of medium IUA in 2000 was randomly distribute in 2000, but this pattern changed into dispersed in 2012.

**Fig 4:**
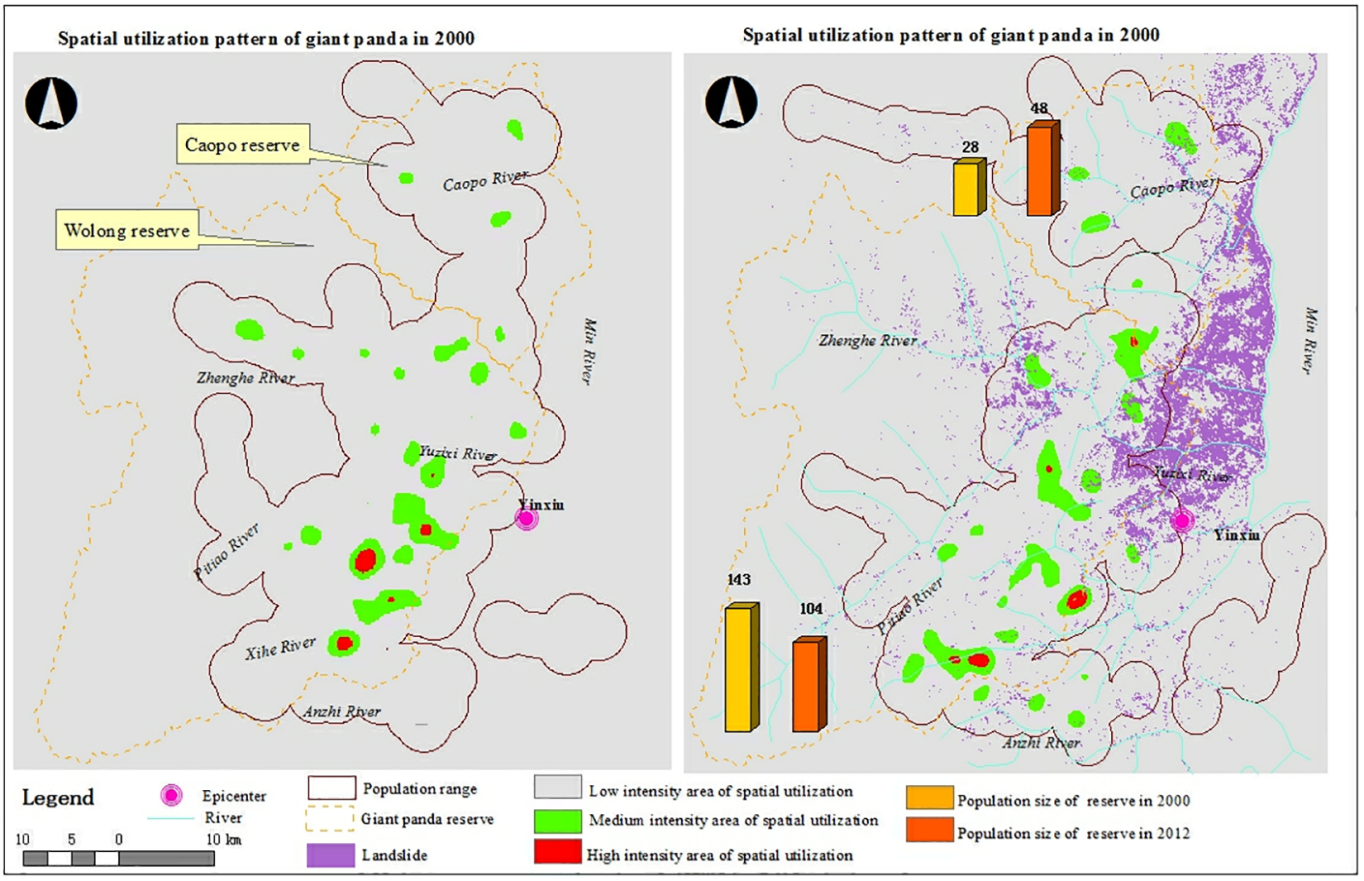
Spatial utilization pattern of the giant panda population in Northern Qionglai Mountains in 2000 and 2012 respectively, Southwest of China.

The medium IUA was in a random spatial pattern (nearest neighbor ratio = 1.10, z-score = 1.31, p = 0.19) in 2000, and a dispersed spatial pattern in 2012 (nearest neighbor ratio = 1.32, z-score = 3.36, p<0.001; Fig 3), indicating a change between the two surveys in the distribution of areas utilized by giant pandas. The number of high IUA was similar between two surveys (5 vs. 4; Fig 4). However, the mean distance to nearest neighbor was significantly longer in 2012 (14.1±1.9km) than in 2000 (6.1±0.9km; t = -7.4, df = 5, p = 0.001). Compared to the total number of encounters, the proportion of the corridor increased 45.76% throughout the study, from 6.14% in 2000, to 8.95% in 2012. The density of giant panda occurrence in the corridor increased 24.6% throughout the study, from 1.26×10^−2^ /ha in 2000 to 1.57×10^−2^ /ha in 2012. In the region near the corridor there were three small sized, medium IUAs in 2000, and one large medium IUA and one high medium IUA located directly within the corridor in 2012 (Fig 4), indicating more frequently using of corridor in 2012.

## Discussion

In the 12 years following the creation of Caopo Nature Reserve, the population size and density of giant pandas increased within its boundaries, and decreased in the adjacent Wolong Nature Reserve. Our results indicate that the performance of Caopo is likely due to the high proportion of suitable habitat available, and large area of connectivity with Wolong. The increase in giant panda occurrences and in the concentration of high intensity utilization areas within the corridor between Wolong and Caopo indicate that this area may have facilitated giant panda dispersal or movement between the reserves. At the very least the additional protected suitable habitat promoted use by pandas in the area. These results indicate that if designed appropriately, new reserves like Caopo, can serve as more than an isolated habitat for local giant panda population but also as an effective part of a broader habitat network. Given the increase in giant panda population over this study, and the relatively high habitat availability and connectivity to another large area of suitable habitat, we consider Caopo a valuable habitat and effective in giant panda conservation at regional scale.

Without genetic or GPS collar data it is difficult to determine how much of the population growth in Caopo was from the reproduction of individuals previously existing in the area, and how much was from the immigration of individuals from nearby habitat. However, we should expect an abundance of suitable habitat to promote population growth through reproduction, and high connectivity enables immigration. The observed steep increase of giant panda population in Caopo (71.4%) cannot be completely explained by natural population growth for several reasons. First, observed population growth of Caopo was far higher than the average growth rate at nationwide (16.8%), provincial (12.8% to 26.4%), and mountain range (20.82%) levels [23], which is unlikely to have resulted from preexisting differences in the fecundity of the populations or in the habitats. Second, Wolong and Caopo are connected nature reserves that share similar habitat structure and keep connecting after massive nature disturbance. With less habitat loss and a relatively high proportion of suitable habitat, Caopo may promote immigration, or alternatively discourage emigration, more than Wolong.

Caopo is the northern distributional limit of giant pandas in the Qionglai Mountains. With respect to the relatively isolated landscape pattern, there is no known source of giant pandas that could immigrate into Caopo except from Wolong. It is unlikely that under similar natural conditions one population would experience such a large decline (-27.3% in Wolong) while the other nearly doubled in size. Ideal free distribution theory [24] and the density dependent dispersal hypothesis [25] may be the mechanism behind the population decline in Wolong and increasing in Caopo. However, future molecular analyses are necessary to test this hypothesis. It is also important to note that differences in the intensity of use or locations of sampling transects between the 2000 and 2012 NGPS introduce a source of uncertainty in the data.

Wolong represents the core giant panda population and habitat in Qionglai, therefore, the factors influencing the local population decline reported here should be carefully considered. Following its inception, the habitat quality and connectivity in Wolong continued to decline due to anthropogenic uses, including timber harvesting, livestock, and agriculture [8, 26]. Additional disturbance from the 2008 earthquake and subsequent reconstruction activities caused a rapid reduction in the availability of suitable habitat within both reserves and in the corridor. Given the more than 40% reduction in suitable that we found between 2000 and 2012, we are not confident that the corridor will function well if such a disturbance happens again. Thus, to better maintain nature reserve network functions it is important to periodically conduct quantitative analyses to identify corridors that may be vulnerable to disturbance or are in need of repair.

Currently, much of the habitat in the giant panda nature reserve network is poorly connected due to natural or human induced barriers, and the availability of suitable habitat for pandas can be relatively low within their borders [27]. Our study emphasizes the importance of effectively utilizing the limited land and funding resources available for conservation by creating additional reserves in strategically located positions. Such reserves may promote natural population growth, or when environmental changes occur within a given range, which might be induced by numerous natural or anthropogenic causes (e.g. disease, bamboo flowering, natural disturbances, climate change, or forest fire), the local populations of endangered species are able to survive. Specifically, we suggest that when screening lands adjacent to a major protected area (potential source population) to determine their priority as a nature reserve, they should: 1) contain a high proportion of suitable habitat, 2) be well connected to the existing nature reserve from both natural and anthropogenic landscape features (corridor), and 3) have a low population density relative to the existing reserve. This criteria is not only applicable to the giant panda reserve network, but should also be considered in the conservation and management of other endangered species facing similar problems.

Furthermore, it is often difficult to estimate the population sizes of forest dwelling wildlife across montane systems due to poor accessibility and visibility. Therefore, many indirect population estimation methods arise, like pellet group count [28], capture-recapture [29], passive trail cameras [30], etc. However, there is no universally applied wildlife population estimation method used at a large scale. Thus, we acknowledge there may be limitations in the giant panda population estimation method utilized. However, currently, it is the only widely applied method for giant pandas (used in the NGPS’s), and is at the base of giant panda conservation.

New nature reserves are continually being created in mountain ranges within the distribution of giant pandas, including the Minshan and Qinling Mountains. However, the reserves in the network are not managed under one universal system, and their management depends on whether they are designated as either national, provincial, city, or county level [16]. Different levels receive varying amounts of financial support and other resources from the government, and thus the ability of wildlife managers to protect the habitat also varies. However, as our results indicate, even lower level nature reserves that contain suitable habitat for giant pandas, like Caopo, can be important to the overall giant panda conservation effort. We urge the central and regional agencies of the Chinese government re-examine the current reserve network and to provide a more universal management system (e.g. national park system) and funding provisions throughout the range of giant pandas.

## Conclusions

Our study illustrates the fact that although the Fourth NGPS reported an increase in both the total population size of giant pandas and available habitat, there remain many regional uncertainties to their continued persistence. New nature reserves have the potential to improve the conservation status of giant pandas, as reported here of Caopo. However, there are still considerable threats to the ongoing conservation success, especially habitat loss and fragmentation induced by anthropogenic disturbances, and the fact that a considerable portion of giant panda habitat is located outside of the nature reserve network [13,31]. As climate change threatens to further disrupt the distributions of pandas and their habitats, it is critical to increase the area protected and connectivity between the reserves [32]. These issues must be fully addressed if we are to ensure giant pandas and the species sympatric with them are to survive in perpetuity.
